# Educational strategies on tooth avulsion for teachers: an intervention study

**DOI:** 10.1590/1807-3107bor-2025.vol39.031

**Published:** 2025-03-10

**Authors:** Thaíssa Chagas FOCHI, Simone TUCHTENHAGEN, Juliane Carla TAUFER, Fernanda Ruffo ORTIZ

**Affiliations:** (a) Atitus-Educação, Passo Fundo, RS, Brazil.; (b) Regional Integrated University of Upper Uruguai and Mission, Erechim, RS, Brazil.

**Keywords:** Tooth Avulsion, Knowledge, Health Education, Clinical Trial

## Abstract

Tooth avulsion is an emergency dentoalveolar trauma, and knowledge of this condition can improve the prognosis of trauma. This study aimed to assess and improve elementary school teachers’ knowledge of tooth avulsion using two educational intervention methods. Data were obtained through a parallel, blinded educational intervention study involving 116 teachers from public and private schools. They participated and completed a structured questionnaire regarding the immediate measures required for dentoalveolar trauma to permanent teeth, storage methods, and the ideal time for the dental element to remain outside the socket. After randomly assigning teachers to the leaflet and video groups, they were provided guidance on dentoalveolar trauma and completed the questionnaire again. Comparison tests, namely chi-square and logistic regression, were performed to assess the post-intervention responses and compare the differences between the groups. The results were interpreted with a significance level of 5% and a 95% confidence interval. Most responses showed a statistically significant difference (p < 0.05), indicating that the interventions improved teachers’ knowledge, except for questions on time and immediate management after dentoalveolar trauma (p > 0.05). There was no statistical difference between the intervention methods, demonstrating that both the leaflets and videos improved teachers’ knowledge (p > 0.05). The results revealed that teachers’ knowledge of tooth avulsion improved regardless of the format of the educational intervention.

## Introduction

Tooth avulsion, a dentoalveolar trauma characterized by the total displacement of the tooth out of the socket, is considered one of the most serious dental injuries.^1^ This condition, which affects children and school-age adolescents, has a 1−11% prevalence among all traumatic injuries to permanent teeth.^1,2^ Tooth avulsion is an emergency situation requiring immediate tooth removal or referral to a dental surgeon.^3,4^ For a good prognosis of the dental element, it is necessary to maintain the vitality of the periodontal ligament,^5,6^ which may be facilitated by the storage medium.^3,4^

Previous studies have shown that teachers have insufficient knowledge of how to properly manage dental injuries and emergency procedures.^5,7-9^ Given the significant amount of time children spend in schools and the possibility of dental injuries occurring during sporting activities due to falls or accidents, it is essential to address this knowledge gap in teachers’ training and education.^7,10^ In cases of dental trauma, the process must be performed successfully from the moment of trauma to referral to a dentist.^11^ Therefore, good case management depends on the teacher’s level of knowledge, enabling early intervention that can improve the chances of a positive prognosis.^12^

Previous research has demonstrated the effectiveness of dentists’ interventions in motivating patients, students, teachers, and parents. Different forms of educational resources, including posters, leaflets, videos, websites, social media, and mobile health apps, are available.^13^ On the one hand, multimedia is a promising medium for patient education.^14^ Videos present complex information visually and engagingly, facilitating comprehension and retention through images, animations, and narration. On the other hand, leaflets are low-cost, easily distributable, and accessible to a wide audience without requiring advanced technology, thus providing information on different types of dental trauma prevention.^15^ Thus, both leaflets and videos are effective in providing information.^14^

Educating and training elementary school teachers are primary strategies dental surgeons must actively and continuously participate in.^16^ However, educational intervention studies involving the Brazilian population, particularly teachers, are scarce. Dental traumatology is an important topic in pediatric dentistry that must be explored more in the community. Furthermore, teachers need to gain knowledge of subjects they can share with their students, parents, and colleagues. This study aimed to assess teachers’ knowledge of tooth avulsion using two educational intervention methods to improve their knowledge. The following are the study’s conceptual hypotheses: a) the findings will reveal whether intervention methods improve teachers’ knowledge, and b) there will be no difference between the two intervention methods.

## Methods

This paper was guided by the Consolidated Standards of Reporting Trials.^17^ The study protocol was registered in the Open Science Framework (https://doi.org/10.17605/OSF.IO/WN5V3) and the Registro Brasileiro de Ensaios Clínicos (REBEC - UTN code: U1111-1305-3263).

### Ethical approval

This study was approved by the Research Ethics Committee of ATITUS Education (CAAE No. 64528222.9.0000.5319). The teachers participated in the study after signing an informed consent form. All schools and education departments agreed to participate in the study.

### Study design

A parallel and blind educational intervention was conducted in 2023 with teachers from public and private elementary schools that had enrolled 12-year-old adolescents. According to the World Health Organization, this is the ideal age range for monitoring oral health in adolescents as they already have permanent teeth.^18^

### Sample

This study was conducted in the cities of Frederico Westphalen and Getúlio Vargas in northern Rio Grande do Sul, Brazil. According to national data, Frederico Westphalen and Getúlio Vargas had 32,627 and 16,602 inhabitants, respectively, in 2023.^19^ This study included elementary schools with adolescents aged 12 years old who consented to participate by signing a free and informed consent form. All schools meeting the eligibility criteria were contacted, and all teachers were invited to participate in the study. Teachers undergoing training and trainees were excluded.

GPower was used for the sample calculation. The a priori calculation was based on a t-test with a difference in means between the independent groups. A two-tailed test was considered with an effect size of 0.5 (medium), a significance level of 0.05, and a statistical power of 0.80. The mean group allocation was 1. The sample size per group was 64, with a minimum of 128. After adding 20% for possible losses or refusals, the final sample size was 154 participants (teachers).

### Data collection

A structured questionnaire consisting of ten closed questions was administered by the researchers in person and completed by the teachers to assess their knowledge of tooth avulsion.

The questions were as follows: 1) “Do you know what an avulsed tooth is?” 2) “Do you know what a dental reimplantation is?”, and 3) “Would you reimplant an avulsed tooth?” The answer options for these three questions were “Yes” and “No.” 4) “What would you do if a student suffered avulsion of a permanent tooth?” The answer options were “I would call the parent or guardian.” (wrong answer), “I would take the child to the dentist immediately.” (wrong answer), “I would take the child to the emergency room.” (wrong answer), and “I would put the tooth back in the socket and run to the dentist.” (right answer).^3,4^ 5) “If the permanent tooth fell on the floor and got dirty, what would you do?” The answer options were “I wouldn’t clean the tooth.” (wrong answer), ” I would clean the tooth with running water.” (right answer), “I would clean the tooth with saline solution.” (wrong answer), and “I would clean the tooth with soap and water.” (wrong answer). 6) “How would you hold the tooth or tell someone to hold it?” The answer options were “Hold it by the crown (right answer), “Hold it at the root.” (wrong answer), and “Hold it anywhere.” (wrong answer). 7) “How long can a permanent tooth be left out of the mouth before being replaced in the socket?” The answer options were “The tooth must be reimplanted immediately or within 1 hour.” (right answer)^6^ and “The tooth must be implanted after more than 2 hours.” (wrong answer). 8) “If reimplantation is not performed, how should the child or teenager take the tooth to the dentist?” The answer options were “The tooth must be transported in an environment with saliva, inside the mouth or in a container.” (wrong answer), “The tooth must be transported in a container containing water.” (wrong answer), “The tooth must be transported wrapped in a piece of gauze.” (wrong answer), and “The tooth must be transported in a container containing milk.” (right answer).^2^ 9) “Have you ever received any information about managing an avulsed tooth?” The answer options were “Yes” or “No.” 10) “Do you think it is important to have an educational program on the management of dental trauma?” The answer options were “Yes” or “No.” The questions were based on previous literature.^1,20-22^ Furthermore, the teachers reported how long they had been teaching (numerical variable).

A pilot study was conducted with nine undergraduate pedagogy students to verify the structure and understanding of the questionnaire. No adjustments or changes were necessary, and participants were not included in the final sample.

After answering Q1, a simple randomization in two blocks (leaflet and video group) was generated by the online program Sealed.^23^ Researcher A (TCF) informed Researcher B (FRO) about the participant allocation sequence over the phone, thus preserving allocation confidentiality. Research B was blind. Subsequently, Researcher A sent the intervention methods to each participant via WhatsApp. Teachers had 15 days to study the educational intervention methods.

Group 1 (G1) received a leaflet, and Group 2 (G2) received a video.^14^ The information in the materials included the procedures to be performed in the case of tooth avulsion and explained the ideal time for the avulsed tooth to remain outside the socket, the storage method, and what to do at the exact moment of the trauma.^1^ The method of delivery was online. This approach can provide a convenient resource for participants to revisit information at home and at any time, enhancing flexibility and making it easily accessible for education. Therefore, teachers can use online resources whenever necessary.^24^

Fifteen (15) days later, the researchers returned to the schools and readministered the questionnaire (Q2) to avoid memory bias^25^. Q2 was the same as Q1, except that Questions 9 and 10 were excluded.

### Outcome

The primary outcome of this study was the improved knowledge of tooth avulsion among teachers. All questions contained only one correct alternative and a categorical variable was considered.

### Statistical analysis

Data were analyzed using descriptive statistics, absolute frequencies, percentages, means, and standard deviations (SD). The answers were divided into incorrect and correct. Chi-squared tests were performed to assess baseline responses, compare intervention methods (leaflets and videos), and compare responses at baseline and post-intervention. Logistic regression analyses were performed to assess post-intervention responses (outcomes) between intervention methods (predictors) adjusted for baseline responses. The results were interpreted with a significance level of 5% and a 95% confidence interval. Statistical analyses were performed using STATA version 14.

## Results

Thirteen public schools and two private schools agreed to participate in the study, resulting in 330 invited teachers. Of them, 160 agreed to participate and completed the initial questionnaire (Q1). Among the participants, 78 received an educational leaflet (G1) and 82 received a video (G2). However, there was a loss of adherence during the intervention phase (second stage), with only 116 participants completing both the pre- and post-intervention questionnaires. Specifically, 51 teachers remained in G1 and 65 remained in G2 (Figure).

**Figure:**
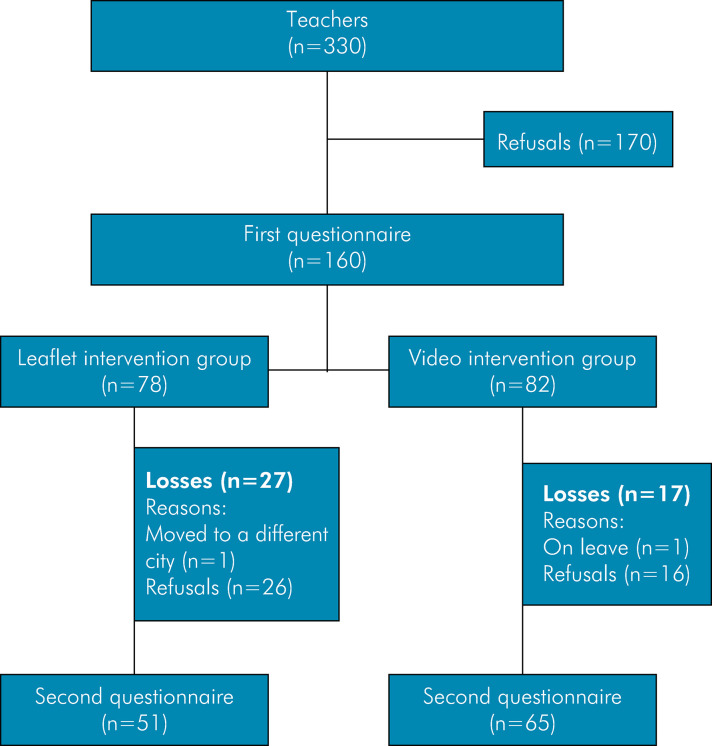
Study of the respondent process flowchart.

Table 1 presents the baseline data, which indicates no statistically significant differences between the participants’ responses in both groups before randomization (p > 0.05), thus confirming that the groups were similar at the start of the study. Among the participants, 91.88% (n = 147) were female with an average teaching experience of 15 years (SD 9.66) (data not shown).

**Table 1:** Descriptive and comparative analysis of baseline responses between Groups 1 and 2.

Variable	Leaflet		Video		p-value*
	(G1)		(G2)		
	n	%	n	%	
1. Do you know what an avulsed tooth is?					0.414
No	64	50.39	63	49.61	
Yes	14	42.42	19	57.58	
2. Do you know what a dental reimplantation is?					0.447
No	37	52.11	34	47.89	
Yes	41	46.07	48	53.93	
3. Would you reimplant an avulsed tooth?					0.353
No	58	46.77	66	53.23	
Yes	20	55.56	16	44.44	
4. What would you do if a student suffered a tooth avulsion of a permanent tooth?					0.087
Wrong answer	73	47.71	80	52.29	
Right answer	5	83.33	1	16.67	
5. If the permanent tooth fell on the floor and got dirty, what would you do?					0.357
Wrong answer	40	45.45	48	54.55	
Right answer	38	52.78	34	47.22	
6 How would you hold the tooth or tell someone to hold it?					0.913
Wrong answer	21	47.73	23	52.27	
Right answer	56	48.70	59	51.30	
7. How long can a permanent tooth be left out of the mouth before being replaced in the socket?					0.267
Wrong answer	14	58.33	10	41.67	
Right answer	57	45.97	67	54.03	
8. If reimplantation is not performed, how should the child or teenager take the tooth to the dentist?					0.381
Wrong answer	59	50	59	50	
Right answer	15	41.67	21	58.33	
9. Have you ever received any information about managing an avulsed tooth?					0.637
No	73	48.03	79	51.97	
Yes	4	57.14	3	42.86	
10. Do you think it is important to have an educational program on the “management of dental trauma”?					0.763
No	3	42.86	4	57.14	
Yes	74	48.68	78	51.32	

*Chi-square test.

Table 2 presents a comparison of the responses between the pre- and post-intervention phases. A statistically significant improvement was observed in the knowledge of avulsion, tooth reimplantation, cleaning, and tooth transfer (p < 0.05), indicating that participants in both groups shifted their responses to the correct alternatives following the interventions. However, no significant changes were detected in questions regarding the timing and immediate actions taken after dental trauma (p > 0.05).

**Table 2:** Comparative analysis between pre-intervention and post-intervention responses.

Variable	Post-intervention				p-value*
	Wrong		Right		
	n	%	n	%	
1. Do you know what an avulsed tooth is?					0.046
Wrong answer	22	23.40	1	4.55	
Right answer	72	76.60	21	95.45	
2. Do you know what a dental reimplantation is?					0.001
Wrong answer	13	23.21	2	3.33	
Right answer	43	76.79	58	96.67	
3. Would you reimplant an avulsed tooth?					0.020
Wrong answer	47	51.65	6	25.00	
Right answer	44	48.35	18	75.00	
4. What would you do if a student suffered a tooth avulsion of a permanent tooth?					0.655
Wrong answer	75	69.44	3	60.00	
Right answer	33	30.56	2	40.00	
5. If the permanent tooth fell on the floor and got dirty, what would you do?					0.007
Wrong answer	26	40.62	9	17.31	
Right answer	38	59.38	43	82.69	
6. How would you hold the tooth or tell someone to hold it?					0.038
Wrong answer	7	22.58	7	8.33	
Right answer	24	77.42	77	91.67	
7. How long can a permanent tooth be left out of the mouth before being replaced in the socket?					0.580
Wrong answer	0	0	2	2.15	
Right answer	14	100	91	97.85	
8. If reimplantation is not performed, how should the child or teenager take the tooth to the dentist?					0.016
Wrong answer	29	34.52	3	10.71	
Right answer	55	65.48	25	89.29	

* Chi-square test.

Table 3 shows the logistic regression analysis comparing post-intervention knowledge (outcome) with the method of intervention, adjusted for baseline responses. The results revealed no statistically significant difference between the two educational methods (p > 0.05), suggesting that both the leaflets and video interventions were equally effective in enhancing teachers’ knowledge of dental trauma prevention.

**Table 3:** Logistic regression analysis between post-test responses (outcome) and intervention methods.

Variable	Methods		p-value*
	Leaflet	Video	
	(G1)	(G2)	
	OR (95% CI)	OR (95% CI)	
1. Do you know what an avulsed tooth is?	1	1.42 (0.56–3.62)	0.455
2. Do you know what a dental reimplantation is?	1	1.28 (0.40–4.00)	0.670
3. Would you reimplant an avulsed tooth?	1	1.26 (0.59–2.70)	0.540
4. What would you do if a student suffered a tooth avulsion of a permanent tooth?	1	0.75 (0.33–1.69)	0.492
5. If a permanent tooth fell on the floor and got dirty, what would you do?	1	0.97 (0.42–2.23)	0.959
6. How would you hold the tooth or tell someone to hold it?	1	0.47 (0.13–1.65)	0.243
7. How long can a permanent tooth be left out of the mouth before being replaced in the socket?	1	–	-
8. If reimplantation is not performed, how should the child or teenager take the tooth to the dentist?	1	0.53 (0.22–1.27)	0.156

OR: Odds ratio; CI: Confidence interval. * Adjusted for baseline responses. Question 7 had no regression results as almost all answers were correct.

## Discussion

This study assessed teachers’ knowledge of tooth avulsion in schoolchildren and compared two intervention methods. Consistent with the literature,^5,7-10,12^ the results showed that teachers had insufficient knowledge of emergency procedures at baseline and that both methods were beneficial and improved teachers’ knowledge.

At the beginning of this study, teachers did not know the meaning of tooth avulsion and how to manage it, showing similar results with other populations such as Saudi Arabia,^22^ India,^21^ and Crotia.^26^ The lack of knowledge regarding this clinical situation negatively impacts the prognosis of avulsed permanent teeth. When it is not feasible to reimplant an avulsed tooth at the site of the accident, it is necessary to immediately refer the students to a dental surgeon for treatment. Moreover, the tooth must be stored in an appropriate solution. A systematic review assessed the most suitable storage and transportation means for an avulsed tooth, and milk was the most recommended because its nutrients keep the periodontal ligament cells viable for 2–6 hours.^1^

Successful reimplantation of an avulsed tooth depends on several factors, and timing is crucial. The extant literature highlights the importance of immediate reimplantation to increase the chances of regeneration of the periodontal ligament, as the elapsed time between avulsion and reimplantation will determine the prognosis of the dental element.^6^ Although the ideal time for a favorable prognosis is immediately, or at most 30 minutes, the tooth can last up to 1 hour as long as it is kept in an appropriate solution.^1^ In this study, almost all participants knew about the timing of reimplantation before the intervention. The same was observed in a cross-sectional study of teachers in Brazil, most of whom sought dental care immediately after dental trauma.^27^

Another variable that did not change before and after the intervention was the immediate management of the trauma. Schools in the cities where this study was conducted usually recommend that parents be contacted immediately in the event of an accident involving a child or an adolescent. Thus, even after the educational interventions, teachers followed the school guidelines and did not change their answers to the correct answer.

Most participants reported that they did not have any knowledge or training regarding the management of avulsed teeth and that they were interested in learning more about this topic. Therefore, educating the public about emergency procedures and improving the prognosis of dental injuries, especially avulsions, are crucial. A systematic review showed that oral health education is effective in improving teachers’ knowledge of various oral health issues.^16^ Furthermore, the literature mentions that flyers, leaflets, and posters are traditional tools for providing broad knowledge. Finaly, online sources are promising tools for educating people, especially those familiar with digital technologies.^28-31^

Thus, the conceptual hypothesis of this study, which indicates that the participants acquired knowledge regardless of the educational intervention method used, was accepted. These results highlight the need for educational programs addressing the management of dental emergencies for the entire population. These programs cover a variety of topics from reimplantation techniques and preservation of avulsed teeth to emergency care protocols in cases of dental trauma. These results can also influence the formulation of public educational policies for basic education teachers, as collaboration between various sectors (e.g., dentistry and education) is essential for promoting awareness and effective action. Furthermore, visual materials (e.g., posters) can be posted at schools to assist with emergency procedures and help inform students of the latest knowledge and protocols regarding oral health.^32^

However, the results of this study must be interpreted with caution because of certain limitations. Although this study used questions adapted from previously published research to develop the questionnaire, they were not validated. At the time of planning and conducting this study, no validated questionnaires on this specific topic were available. To ensure that the content aligned with established best practices, the questionnaire was based on recognized guidelines by the Association of Dental Traumatology for managing traumatic dental injuries. Moreover, as questionnaires were used, memory bias and participants’ understanding may have influenced the results. To overcome this, they were re-administered after a certain period, and methods for contacting researchers were presented to resolve queries. Despite their limitations, questionnaires have been demonstrated to be a valid and important method for conducting research, both nationally and internationally.^26,33^ Another limitation of this study was the loss to follow-up, as some participants withdrew before completing the second questionnaire. Despite the researchers’ consistent efforts, including regular communication and providing detailed information to maintain engagement, participant attrition persisted. This is a common challenge in intervention studies as participants may experience scheduling conflicts, lose interest, or encounter unforeseen circumstances that prevent them from completing all phases of the research. There was also a lack of encouragement from school leaders. Nevertheless, loss to follow-up is an expected limitation in studies of this nature, as previously documented in similar research.^34^However, this study had the statistical power to detect differences.

Despite these limitations, we believe that the results are valid, indicating that the teachers’ knowledge improved after educational intervention. The results are consistent with those of a scoping analysis, which showed that several studies reported substantial gains in knowledge, regardless of the participants and the intervention tools, implying that interventions are important and effective.^28^Moreover, this study contributes significantly to the community by establishing important connections between education and dentistry and offering valuable insights into methods to enhance knowledge among key professionals who may be the first responders in dental emergency situations.

In conclusion, this study shows that teachers’ knowledge of tooth avulsion improved after educational interventions with no differences between the methods used, demonstrating their effectiveness.
